# Baicalin attenuates bleomycin-induced pulmonary fibrosis via adenosine A2a receptor related TGF-β1-induced ERK1/2 signaling pathway

**DOI:** 10.1186/s12890-016-0294-1

**Published:** 2016-09-23

**Authors:** Xiaoying Huang, Yicheng He, Yanfan Chen, Peiliang Wu, Di Gui, Hui Cai, Ali Chen, Mayun Chen, Caijun Dai, Dan Yao, Liangxing Wang

**Affiliations:** Division of Pulmonary Medicine, First Affiliated Hospital of Wenzhou Medical University, Key Laboratory of Heart and Lung, Wenzhou, Zhejiang 325000 China

**Keywords:** Pulmonary fibrosis, Baicalin, Adenosine A2a receptor, Transforming growth factor-β1, Extracellular signal regulated kinase1/2

## Abstract

**Background:**

Baicalin has been reported to have anti-fibrosis effect; however, its mechanism still remains to be elucidated. Adenosine A2a receptor (A2aR) is a novel inflammation regulator, and transforming growth factor-β1 (TGF-β1)-induced extracellular signal regulated kinase1/2 (ERK1/2) signaling pathway plays an important role in idiopathic pulmonary fibrosis (IPF). This study was to explore the relationship of A2aR and TGF-β1-induced ERK1/2 in bleomycin (BLM)-induced pulmonary fibrosis in mice, and to investigate whether A2aR mediate the anti-fibrosis effect of Baicalin on BLM-induced pulmonary fibrosis.

**Methods:**

The A2aR−/− and A2aR+/+ mice were respectively divided into three groups: control group, model group, baicalin group. Pulmonary fibrosis was induced in mice of model groups by intratracheal instillation of bleomycin, and baicalin was administered in mice of baicalin groups daily for 28 days. Histopathological and ultrastructural changes of lung tissues were evaluated. Lung coefficient and the levels of hydroxyproline (HYP) in lung tissues were measured at the same time. The levels of serum TGF-β1 were measured by ELISA. The expression of TGF-β1, ERK1/2, p-ERK1/2 and A2aR were detected by western blot and immunohistochemical staining techniques.

**Results:**

Severe lung fibrosis was observed in the bleomycin-treated mice on day 28. The histopathological findings and collagen content of lung tissues were much severer/higher in A2aR−/− mice than in A2aR+/+ mice. We also showed that TGF-β1 and p-ERK1/2 were upregulated in bleomycin-treated mice and expressed higher in A2aR−/− mice compared to A2aR+/+ mice. Besides, bleomycin-treated A2aR+/+ mice had increased A2aR level in lungs. However, long-term treatment with baicalin in A2aR−/− and A2aR+/+ mice significantly ameliorated the histopathological changes in lungs. Moreover, Increased TGF-β1 and p-ERK1/2 expressions in bleomycin-treated A2aR−/− and A2aR+/+ mice were obviously diminished by baicalin. The baicalin-treated A2aR−/− mice had severer lung fibrosis and higher expressions of TGF-β1 and p-ERK1/2 than A2aR+/+ mice. Baicalin has also upregulated the expression of A2aR in A2aR+/+ mice.

**Conclusions:**

Genetic inactivation of A2aR exacerbated the pathological processes of bleomycin-induced pulmonary fibrosis. Together, baicalin could inhibit BLM-induced pulmonary fibrosis by upregulating A2aR, suggesting A2aR as a therapeutic target of baicalin for the treatment of pulmonary fibrosis.

## Background

Idiopathic pulmonary fibrosis (IPF) is a chronic progressive interstitial lung disorder with poor prognosis [1]. It’s characterized by the proliferation of fibroblasts and deposition of extracellular matrix (ECM) proteins, which causes distorted lung architecture and function as well as makes breathing increasing difficult, and finally leads to respiratory failure [2, 3]. Over 5 million people worldwide are affected by IPF with a mean survival time of nearly 3 years [4]. But the mechanism of this disease is still unclear. It includes various pathogenesis, such as inflammation, immune mechanisms, epithelial mesenchymal transition (EMT) and oxidative stress [3, 5–7]. Therefore, no medication can effectively cure this disease and alternative approaches for this unmet medical need are of interest.

Adenosine is an endogenous intracellular purine nucleoside [8]. It exerts various physiological effects by engaging different high-affinity adenosine receptors, among which A2aR is an anti-inflammatory adenosine receptor [9]. A2aR plays a protective role in a variety of diseases. It is reported that activation of A2aR led to attenuation of pulmonary hypertension and lung injury [10, 11]. What’s more, Pharmacology study showed that A2aR agonist, CGS21680 could effectively inhibit lung inflammation that may lead to fibrosis [12], which proves evidence that A2aR, as an endogenous protective factor, plays an important role in inhibiting pulmonary fibrosis.

TGF-β1 is one of the cytokines and plays a significant part in regulating inflammation, cell growth and tissue fibrosis [13]. Moreover, TGF-β1 plays a very important role in the proliferation of lung fibroblasts and promotes the production of ECM. TGF-β1 induces alveolar epithelial to mesenchymal transition and stimulates alveolar epithelial cells (AECs) to convert into myofibroblasts [14], which was the probable mechanism of pulmonary fibrosis (PF). ERK, a member of Mitogen-activated protein kinase (MAPK) superfamily, includes two subtypes: ERK1 and ERK2. They play an important role in the process of cell proliferation and differentiation by regulating the expression of inflammatory mediators and cytokines [15]. In recent years, some evidence has shown that the ERK1/2 signaling pathway has a close link with the TGF-β1 in rat or human lung fibroblasts [16, 17]. It has been reported that EMT is also the pathogenesis of renal interstitial fibrosis, and A2aR activation attenuated the expression of profibrotic mediators TGF-β1 in renal [18]. Therefore, we hypothesize that the knockout of A2aR may aggravate pathological changes of PF by synthesising of ECM via up-regulating TGF-β1 and ERK1/2. Conversely, activation of A2aR may suppress the exacerbation of PF, suggesting that stimulation of A2aR might exert significant protective effect on PF.

Baicalin is a flavonoid compound purified from Scutellaria baicalensis with a wide variety of anti-oxidative, anti-inflammatory, anti-infection and anti-tumor functions [19–22]. As a traditional Chinese medicine with high value and widespread efficacy, baicalin has been used in applications of pulmonary arterial hypertension (PAH), hepatic cancer, acute lymphocytic leukemia, and so on [22–24]. A recent study showed that baicalin suppressed the hepatic fibrosis-induced increase in TGF-β1 levels [25]. Although baicalin has been found to exerted therapeutic effects on rats with lung fibrosis induced by bleomycin and to inhibit fibrosis through multiple pathways [26], there are only few reports concerning the mechanism of baicalin on PF.

In this research, we aimed to explore the essential role of adenosine A2a receptor in a BLM-induced pulmonary fibrosis model using the A2aR KO mice, and investigated whether baicalin protected against pulmonary fibrosis via the TGF-β1-induced ERK1/2 signaling pathway.

## Methods

### Reagents

Baicalin was obtained from Sigma (St. Louis, MO, USA) and dissolved in dimethyl sulfoxide, then diluted by sterile saline. Bleomycin hydrochloride was obtained from Nippon Kayaku (Co, Tokyo, Japan). Hydroxyproline (HYP) assay kits were obtained from Nanjing Jiancheng Biochemical Institute (Nanjing, China). Mouse TGF-β1 enzyme linked immunosorbent assay (ELISA) kit was purchased from R&D (Minneapolis, MN, USA). The rabbit antibodies against ERK1/2, phospho-ERK1/2, and GAPDH were purchased from Cell Signaling Technology (Beverly, MA, USA). The mouse antibodies against TGF-β1 and A2aR were purchased from Abcam (Cambridge, UK). Peroxidase conjugated goat anti-rabbit immunoglobulin G (IgG) and Peroxidase conjugated goat anti-mouse immunoglobulin G (IgG) was purchased by Boster (Wuhan, China). DAB kit and Polink-2 plus Polymer HRP Detection System were provided by ZSGB BIO (Beijing, China). SuperSignal (R) West Femto Maximum Sensitivity Substrate and BCA Protein Assay kit were provided by Thermo Fisher Scientific (Waltham, MA, USA).

### Animals

The A2aR knockout mice were purchased from the Jackson laboratory and were established on the Balb/c strain background. Wild type (WT) Balb/c mice were purchased from Slac Laboratory Animal Company. KO mice were crossed with WT mice to get the heterozygous mice, then the heterozygous mice were crossed with each other to get the KO mice and WT mice. A2AR homozygous KO mice and WT Balb/c mice were bred at the Laboratory Animal Centre of Wenzhou Medical University. Homozygous (A2AR−/−: *n* = 24) and the corresponding wild-type (A2AR+/+: *n* = 24) mice (male, 6–8 weeks old, and weight 17–20 g) were used in this study. Animal housing and experimental protocols were approved by the Animal Ethical Committee of Wenzhou Medical University. The mice were housed in a specific pathogen-free room with 12 h light/dark cycle, controlled temperature (23 ± 2 °C) and humidity (60 ± 10 %). Mice were free access to food and water. All procedures of our experiments were in the guide for the Care and Use of Laboratory Animals published by the US National Institute of Health. Our study also adhered to the ARRIVE guidelines.

### Experimental protocols

The mice were randomly divided into six groups (8 mice/group): WT normal control group (WN), WT BLM model group (WM), WT baicalin group (WB), A2aR KO normal control group (AN), A2aR KO BLM model group (AM), A2aR KO baicalin group (AB). Mice were anesthetized with 1 % sodium pentobarbital (50 mg/kg) by intraperitoneal injection. Then, 5U/kg of bleomycin in sterile 0.9 % NaCl was intratracheally injected into the mice of WM, WB, AM and AB groups to induce pulmonary fibrosis [27]. The mice of WN and AN groups were given the same volume of sterile saline instead of BLM. From day 1 after intratracheal injection, daily intraperitoneal injection of baicalin (120 mg/kg) was administered to the mice in WB and AB groups. The mice in other groups received sterile saline with dimethyl sulfoxide. The DMSO was used to dissolve the baicalin, and the concentration of DMSO was diluted by sterile saline to 0.1 % in the control and drug-containing solution. Each group was given 0.0005 ml DMSO per day. Their lung tissues and serum were harvested after being scarified on the 28th day.

### Measurement of lung coefficient and hydroxyproline (HYP)

The body weight of mice was measured before being sacrificed. After sacrificing the mice, the lung was excised from body and washed with PBS, then measured weight. The severity of lung injury was assessed by counting lung coefficient (lung weight (mg)/body weight (g) Ratio). The levels of HYP reflected the collagen content and were determined by hydroxyproline (HYP) assay kits according to the manufacturer’s instructions. The absorbance of each well was read at 550 nm. The results were calculated by using the standard formula of hydroxyproline.

### Histopathologic and immunohistochemical staining

The lower lobe of the right lung was fixed in 4 % paraformaldehyde overnight, dehydrated in a graded ethanol series, then embedded in paraffin, and cut to 5um thick. Hematoxylin and eosin (H&E) staining, Masson’s trichrome staining and immunohistostaining were performed on 5 um-thick paraffin-embedded sections. The primary antibodies used for immunohistochemistry: anti-TGF-β1 (diluted 1:50 with PBS), anti-p-ERK1/2 (diluted 1:400 with PBS), anti-A2aR (diluted 1:200 with PBS). The level of histopathological changes and positive stained area in pulmonary was observed by a light microscope. 5 different fields under magnification of 400× were chosen randomly from each section and manufactured one sections for each mice, 8 mice for each group. There were 40 fields for each group. Image-Pro Plus 6.0 (Media Cybernetics, USA) was used to measure the integrated optical density (IOD) of positive stained cells.

### Ultrastructural examination of interstitial tissues of lung

The sample (1 mm^3^) was taken from the right lower lung peripheral tissue, fixed in 2.5 % glutaraldehyde and 1 % osmium tetroxide, dehydrated with acetone, and then embedded in EPON 812. The fixed tissue pieces were cut into ultrathin sections by LKB-V-type ultramicrotome. The thin sections were stained with uranyl acetate and lead citrate, and then examined under the transmission electron microscopy (H-7500, Hitachi, Japan).

### Cytokine assay

Concentration of TGF-β1 in serum was measured by ELISA kits according to the manufacturer’s instructions. The optical density of each sample was read at 450 nm. The results were calculated by using the linear regression equation of standard curve.

### Western blotting analysis

The lung tissues were homogenised in RIPA lysis buffer containing PMSF. Then the homogenates were centrifuged at 12,000 rpm for 30 min at 4 °C. The supernatants containing total protein were collected. The protein concentration was determined by the BCA protein assay. Equal amounts of proteins (20 μg) per lane were separated by 12 % sodium dodecyl sulfate-polyacrylamide gel (SDS-PAGE) and blotted to polyvinylidene difluoride (PVDF) membranes, then the membranes were blocked for 1 h in 5 % nonfat milk and incubated overnight at 4 °C with primary antibodies against TGF-β1(1:500), ERK1/2(1:1000), phospho-ERK1/2(1:2000), A2aR(1:1000) and GAPDH(1:5000), respectively, followed by the peroxidase conjugated goat anti-rabbit or anti-mouse IgG(1:10,000) for 1 h. Subsequently, Peroxidase labeling was visualized via enhanced chemiluminescence (ECL) reagents. Images were detected and analysed by Image Lab program (Bio-Rad Laboratories, Hercules, CA, USA).

### Statistical analysis

The data were analysed by using SPSS software version 20.0 (IBM, Somers, NY, USA) and expressed as the mean ± standard deviation (SD). The comparison between 2 groups was analyzed by Student’s *t*-test. The comparison among more than 2 groups was analyzed by one-way ANOVA, followed by post hoc comparison with LSD test (equal variances assumed) or Dunnett’s T3 test (equal variances not assumed). Values of *P* < 0.05 were considered to be statistically significant.

## Results

### A2aR and baicalin reduced the lung coefficient and HYP of the BLM mouse model

**Fig. 1:**
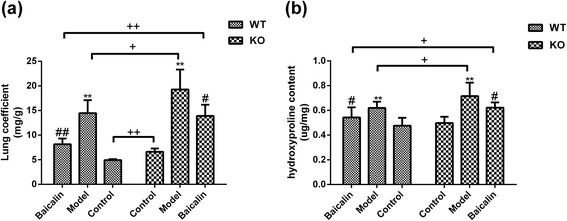
A2aR and baicalin reduced the lung coefficient and HYP of the BLM mouse model. **a** Comparison lung coefficient in each group. **b** Comparison HYP content of mice in each group. Data were expressed as mean ± standard deviation (SD), ** *p* < 0.01 vs control group, ## *p* < 0.01 vs model group, # *p* < 0.05 vs model group, ++ *p* < 0.01 between WT and KO groups, + *p* < 0.05 between WT and KO groups; *n* = 8

As shown in Fig. 1a, the lung coefficien of model groups elevated significantly compared with the control groups in WM and AM groups (*p* < 0.01 and *p* < 0.01, resp), and significantly decreased after baicalin treatment in WB and AB groups (*p* < 0.01 and *p* < 0.05, resp). Besides, the lung coefficien was respectively higher in AN, AM, AB groups than in WN, WM, WB groups (*p* < 0.01, *p* < 0.05, and *p* < 0.01, resp). Figure 1b showed that the HYP contents increased significantly after BLM administration in WM and AM groups (*p* < 0.01 and *p* < 0.01, resp), and significantly reduced by baicalin in WB and AB groups (*p* < 0.05 and *p* < 0.05, resp). Similarly, the content of HYP in AM and AB groups was obviously higher, compared with WM and WB groups (*p* < 0.05 and *p* < 0.05, resp). However, there was no significant difference between AN group and WN group.

### A2aR and baicalin alleviated pulmonary histopathological changes in the BLM mouse model

**Fig. 2:**
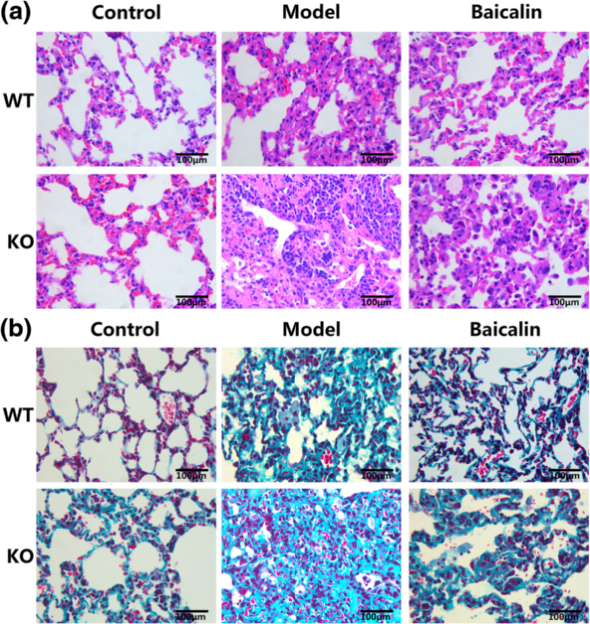
A2aR and baicalin alleviated pulmonary histopathological changes in the BLM mouse model. **a** Images of H&E of pulmonary tissue sections (light microscopy, ×400). Control groups show the normal interalveolar septum; model groups indicate widespread interalveolar septum thickness; baicalin groups exhibit reduced tissue injury. And the histopathological changes in KO mice are more obvious. **b** Images of Masson’s trichrome staining of pulmonary tissue sections (light microscopy, ×400). Control groups show no obvious collagen fibers deposition; model groups indicate an abundance of collagen deposition (blue-stained); baicalin groups exhibit reduced collagen fibers deposition. The deposition of collagen fibers in KO mice is more obvious. *Scale bars* represent 100 μm

HE and Masson’s staining were performed to observe the histopathological changes in lung. The lung of mice in WN group showed no abnormal histological changes (Fig. 2). However, in AN group, the HE staining in the sections revealed the slight thickening of alveolar septum, and the Masson’s staining showed that there was a small amount of collagen depositing in pulmonary interstitial. The model groups indicated significant pathological changes in sections with HE staining, e.g. widespread interalveolar septum thickness, inflammatory cells infiltration and alveolar hemorrhage; and the Masson’s staining indicated an abundance of collagen deposition. Besides, the degree of fibrosis in AM group is the most serious. Baicalin could attenuate the pathological changes induced by BLM, and the effect on WT mice is better than on KO mice.

### A2aR and baicalin ameliorated pulmonary ultrastructural changes in the BLM mouse model

**Fig. 3:**
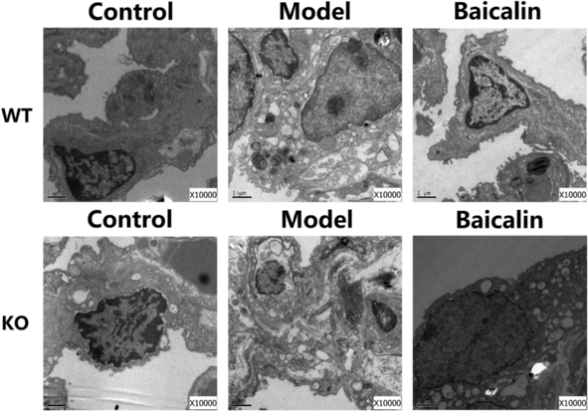
A2aR and baicalin ameliorated pulmonary ultrastructural changes in the BLM mouse model. The ultrathin sections were observed under a Hitachi H-7500 transmission electron microscopy (10,000×). Control groups show little change in ultrastructure; model groups indicate destruction of alveolar epithelial cells and deposition of collagen fibers; baicalin groups exhibit obvious ultrastructural improvement. The ultrastructure of KO mice is more severely damaged. *Scale bars* represent 1 μm

The transmission electron microscopy was used to observe the pulmonary ultrastructural changes. Similar with the results of the pathological sections, there was little change in ultrastructure of WN group, and the interalveolar septum was normal, the junction between cells was tight. Compared with the WN group, there were slight karyopyknosis and vacuolations in the type I alveolar epithelial cells of the AN group (Fig. 3). After BLM administration, the WT model groups showed that type II alveolar epithelial cell became hyperplastic, with production of numerous vacuolations and lamellar bodies, deposition of collagen fibers and the swelling of organelles. Moreover, the AM group indicated the highest number of collagen fibers in the interalveolar septum, with markedly condensation of chromatin, disordered cell structure and deformed basement membrane. After the treatment of baicalin, the ultrastructural improvement of WB group was the most significant, the structure of type I alveolar epithelial cells was complete, and there was no obvious collagen fibers depositing in interalveolar septum. However, the effect of baicalin on KO mice was worse than on WT mice. The number of lamellar bodies in type II alveolar epithelial cells decreased, but there were still vacuolations and collagen fibers in cells.

### A2aR and baicalin attenuated TGF-β1 expression in the BLM mouse model

**Fig. 4:**
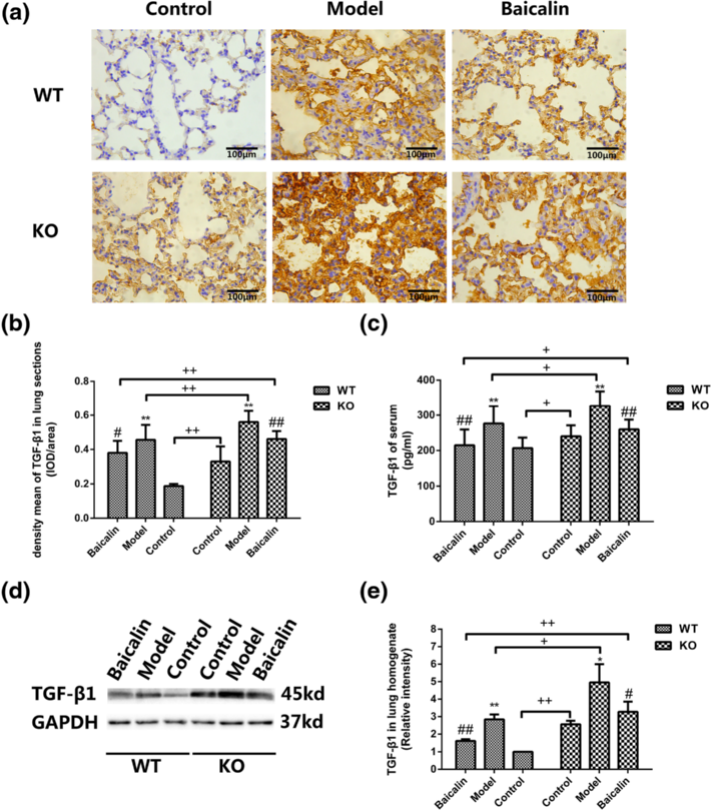
A2aR and baicalin attenuated TGF-β1 expression in the BLM mouse model. **a** Images of TGF-β1 immunohistochemistry in lung tissue sections. Magnification ×400, *scale bars* represent 100 μm. **b** Quantitative analysis of TGF-β1 protein by immunohistochemistry. **c** TGF-β1 content (pg/ml) of serum (ELISA) (*n* = 8). **d** Images of TGF-β1 in lung homogenate (western blot). **e** Quantitative analysis of TGF-β1 in lung by western blot. GAPDH was used as internal control (*n* = 3). Data were expressed as mean ± SD. ** *p* < 0.01 vs control group, * *p* < 0.05 vs control group, ## *p* < 0.01 vs model group, # *p* < 0.05 vs model group, ++ *p* < 0.01 between WT and KO groups, + *p* < 0.05 between WT and KO groups

We measured the expression of TGF-β1 in serum and lung by ELISA, immunohistochemical staining and Western blotting. Examination by ELISA revealed that TGF-β1 in serum was markedly upregulated in the model groups (*p* < 0.01 and *p* < 0.01, resp). On the contrary, baicalin efficiently reduced TGF-β1 expression (*p* < 0.01 and *p* < 0.01, resp) (Fig. 4c). Compared with WT mice, the concentration of TGF-β1 in KO mice was higher (*p* < 0.05, *p* < 0.05, and *p* < 0.05, resp). Consistent with the ELISA, the results of immunohistochemical staining and western blot also indicated the differences in TGF-β1 expression in WT and A2aR KO mice (Fig. 4a, b, d and e).

### A2aR and baicalin attenuated ERK1/2 phosphorylation in the BLM mouse model

**Fig. 5:**
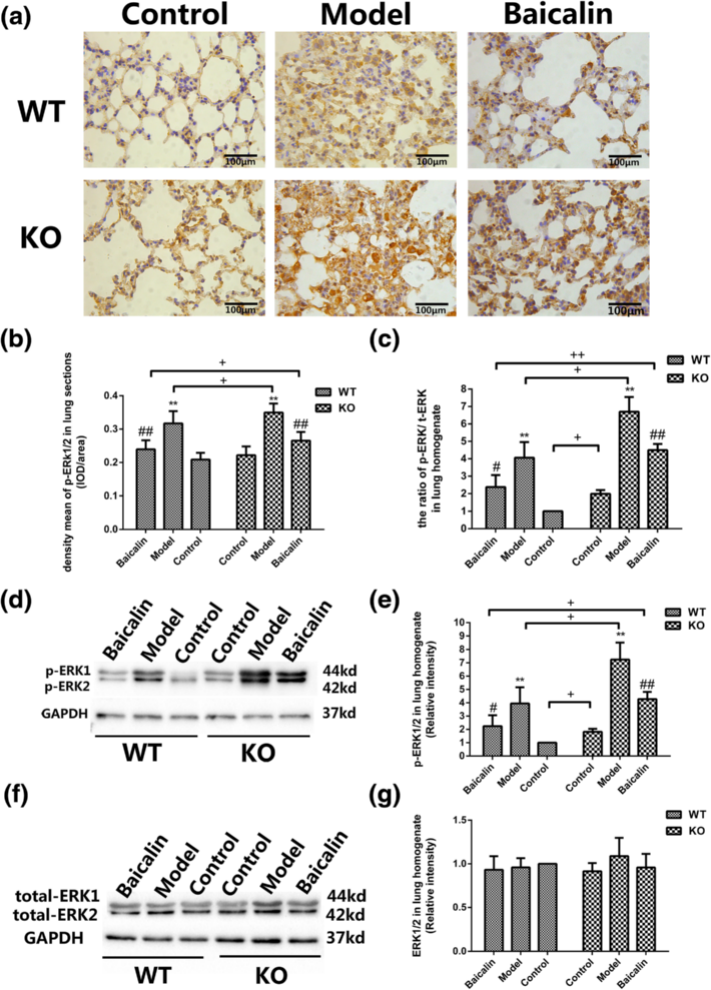
A2aR and baicalin attenuated ERK1/2 phosphorylation in the BLM mouse model. **a** Images of p-ERK1/2 immunohistochemistry in lung tissue sections. Magnification ×400, *scale bars* represent 100 μm. **b** Quantitative analysis of p-ERK1/2 protein by immunohistochemistry. **c** The protein expression ratios of phospho-ERK1/2 to total-ERK1/2. **d** Images of p-ERK1/2 in lung homogenate (western blot). **e** Quantitative analysis of p-ERK1/2 in lung by western blot. **f** Images of total-ERK1/2 in lung homogenate (western blot). **g** Quantitative analysis of total-ERK1/2 in lung by western blot. GAPDH was used as internal control (*n* = 3). Data were expressed as mean ± SD. ** *p* < 0.01 vs control group, ## *p* < 0.01 vs model group, # *p* < 0.05 vs model group, ++ *p* < 0.01 between WT and KO groups, + *p* < 0.05 between WT and KO groups

In both WT and KO mice, western blot indicated an obvious increase of phospho-ERK1/2 in model groups (*p* < 0.01 and *p* < 0.01, resp) and a remarked reduction of phospho-ERK1/2 in baicalin groups (*p* < 0.05 and *p* < 0.01, resp), and there were significant differences between the WN, WM, WB groups and AN, AM, AB groups (*p* < 0.05, *p* < 0.05, and *p* < 0.05, resp) (Fig. 5d, e). But the total ERK1/2 was not significant in each group (Fig. 5f, g). The ratio of phospho-ERK1/2 to total ERK1/2 accorded with the phospho-ERK1/2 (Fig. 5c). The immunohistochemistry of phospho-ERK1/2 revealed the same result, except that there was no significant difference between normal groups (Fig. 5a, b).

### Baicalin increased A2aR expressions in the BLM mouse model

**Fig. 6:**
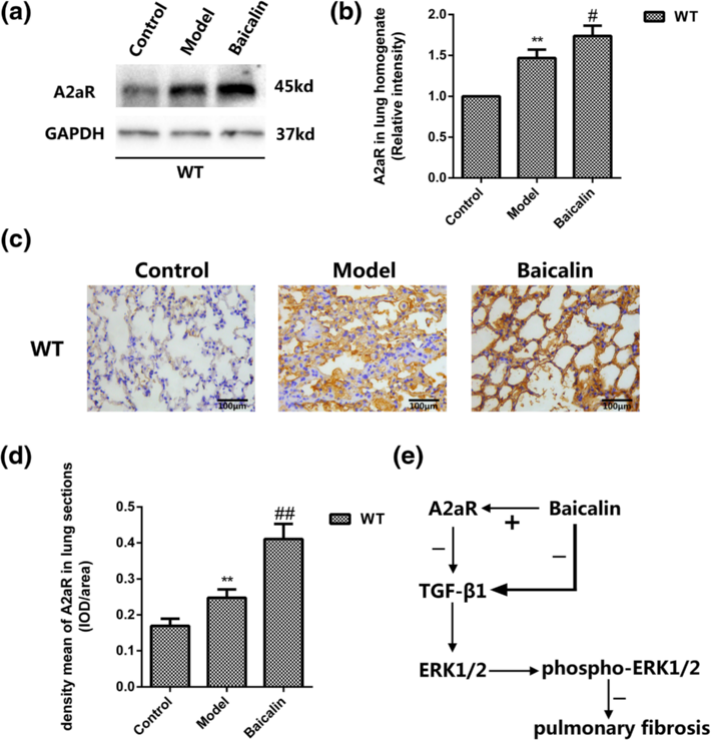
Baicalin increased A2aR expressions in the BLM mouse model. **a** Images of A2aR in lung homogenate (western blot). **b** Quantitative analysis of A2aR in lung by western blot. GAPDH was used as internal control (*n* = 3). **c** Images of A2aR immunohistochemistry in lung tissue sections. Magnification ×400, *scale bars* represent 100 μm. **d** Quantitative analysis of A2aR protein by immunohistochemistry. **e** The signaling pathway was demonstrated in this experiment. Data were expressed as mean ± SD. ** *p* < 0.01 vs control group, ## *p* < 0.01 vs model group, # *p* < 0.05 vs model group

We tested the effects of baicalin on A2aR protein expressions of WT mice after bleomycin administration by immunohistochemical staining and western blotting, and the results suggested bleomycin increased A2aR expression in WM group compared with WN group (*p* < 0.01, and *p* < 0.01, resp). Besides, baicalin treatment enhanced A2aR expression in WB group (*p* < 0.01, and *p* < 0.05, resp) (Fig. 6a, b, c).

## Discussion

Jia et al. have already found baicalin could attenuate bleomycin-induced pulmonary fibrosis in rats [26]. But in our study, we proved that baicalin alleviated pulmonary fibrosis via A2aR related TGF-β1 induced ERK1/2 signaling pathway.

IPF is a progressive and terminal disease with high mortality. Bleomycin-induced lung fibrosis animal model is widely used to investigate IPF, which is characterized by the destruction of the lung architecture and collagen deposition in the lungs [28]. Thus, the increase in lung coefficient and HYP as well as the obvious changes in pulmonary histopathology and ultrastructure of model groups indicated success in establishing the model of IPF.

Although the mechanisms underlying pulmonary fibrosis remains elusive, chronic persistent inflammation and epithelial mesenchymal transition (EMT) have been considered the two important pathogenic events of IPF [29]. Therefore, inhibition of inflammation or EMT may be an efficient therapeutic strategy for IPF. Adenosine, an endogenous intracellular purine nucleoside, is at low concentrations extracellularly under normal conditions, but released increasingly by cells during inflammation and tissue destruction [8, 30]. It regulates cellular function by interacting with a family of 4G protein–coupled receptors, A1R, A2aR, A2bR, and A3R [31]. It is well known that A2aR is expressed on macrophages, dendritic cells, T cells, B cells, epithelial cells and considered as a novel endogenous modulator affecting the inflammation process and tissue repair [32]. Zhou detected that A2aR was down-regulated in severe IPF patients [33], but in our study, an increased A2aR expression was determined in lung tissue in BLM-treated WT mice, which may be a provisional compensatory increase and related to the course of IPF, we will clarify the detailed change of A2aR in the future. Scheibner KA et al. found A2aR-null mice are more susceptible to bleomycin-induced lung injury [12], demonstrated that A2aR plays an important role in inhibiting pulmonary fibrosis of mice. But Scheibner’s study only observed histopathologic changes of lung tissue on 7 days after the mice were treated with BLM, at that time, pulmonary fibrosis was not so obvious. In our study, lung coefficient and HYP was significantly higher in KO mice than in WT mice. The structure of lung, which was observed under the light microscope and transmission electron microscopy, have been found changed more obviously in KO mice than in WT mice. All these findings proved that the degree of pulmonary fibrosis in A2aR−/− mice was more serious and A2aR played a protective role in IPF, which is consistent with other studies. Noteworthy, the difference in lung coefficient between WN group and AN group was significance, and HE and Masson’s staining of AN group showed slight fibrosis.while there was no significant difference in HYP content between the two groups. We infer that it is because the deficiency of A2aR would weaken the body’s resistance to external stimulation (saline), thus causing damage to lung which led to slight fibrosis rather than obvious pulmonary fibrosis.

EMT is a hot research topic at present. Many studies have indicated that EMT has a close relationship with pulmonary fibrosis. In the process of EMT, type II alveolar epithelial cell is considered to be the primary cell which repairs alveolar epithelium after injury, and it can transform into type I alveolar epithelial cell, fibroblast and myofibroblasts after injury. Finally the deposition of ECM leads to the development of IPF [34–36]. TGF-β1 is thought to drive the cardinal processes to the initiation and progression of lung fibrosis, including inflammatory response, epithelial cell apoptosis, EMT, fibroblast proliferation, and collagen deposition [37]. In our model, the immunohistochemistry and western analysis shown the expression of TGF-β1 increased to varying degrees after baicalin administration to both WT and KO mice.

Mitogen-activated protein kinase (MAPK) superfamily (ERK, p38, and JNK) is an important family of enzymes associated with cell membrane receptors and regulative targets, and ERK1/2 are members of the MAPK family. ERK1/2 signaling pathway is the most thoroughly studied signal transduction pathways. They can be activated by a series of cell growth factors and become phospho-ERK1/2, then transmits extracellular signals from the cell surface to the nucleus, which contributes to the proliferation and differentiation of cells, such as proliferation of lung fibroblasts and production of extracellular matrix (ECM). Researchers have proved that the ECM process may be achieved partly via the activation of TGF-β pathway, involving the TGF-β1-induced MAPK pathway [38, 39]. Taken together, the similarly upregulation of the protein level of pulmonary ERK1/2 in BLM-treated mice observed in this study may be explained by the above mechanism. However, there is no direct report about the relationship between A2aR and TGF-β1-induced ERK1/2 pathway in pulmonary fibrosis. Our data shown a significantly increased protein expression of TGF-β1 and phospho-ERK1/2 in KO model mice, which illustrated that A2aR could down-regulate the TGF-β1-induced ERK1/2 pathway in lung fibrosis for the first time. From another point of view, these results confirmed the protective effect of A2aR on pulmonary fibrosis. It is interesting to note that in the western blot graph of p-ERK1/2, the increase degree of p-ERK1 in WM group seems higher than that of p-ERK2, but no obvious difference was found in the rest of groups between p-ERK1 and p-ERK2. We speculate that there are two probable reasons: the first reason is that it was caused by the differences between individual mice; the second reason is that the pathway of ERK1 was indeed more associated with IPF than ERK2. Though the underlying mechanism is still unclear, we will perform further researches to clarify it.

A variety of clinical methods are used to treat IPF, such as corticosteroids, azathioprine, pirfenidone, anticoagulants, tyrosine kinase inhibitors and N-acetyl-cysteine [40]. Corticosteroids has been widely used as the prescribed treatment of IPF in clinic for a long time, but the efficacy of this treatment is not satisfied and its side effects are severe [41, 42]. And the effect of other drugs was not significant. So it is critical and urgent to discover a drug that has a good effect and few side effects. Baicalin, a pharmacologically bioactive components purified from Scutellaria baicalensis, possesses diverse pharmacological activities. Several studies demonstrated that baicalin exerted a therapeutic effect on hepatic fibrosis [43, 44]. Gong LK et al. discovered Feitai was useful in the treatment of pulmonary fibrosis, and Feitai is a Chinese composite formula comprising Scutellaria baicalensis [45]. Baicalin was also reported to inhibit the expression of TGF-β1 in pulmonary arterial hypertension [46]. However, whether baicalin would inhibit TGF-β1-induced MAPK pathway via adenosine A2a receptor has not been investigated. In our experiment, baicalin was found to be effective in reducing the expression of TGF-β1 and phospho-ERK1/2 in pulmonary fibrosis, thus partially inhibiting epithelial mesenchymal transition (EMT), which may be a critical mechanism via which baicalin protects mice with PF. Moreover, A2aR expressed the highest after baicalin treatment, suggesting that baicalin exerted effects on PF through A2aR. Consistently, this study showed that baicalin activated A2aR and the activation of A2aR suppressed TGF-β1 activation, which down-regulated ERK1/2 expression (Fig. 6d).

## Conclusions

In summary, baicalin is effective in inhibiting histopathological and ultrastructural damages in BLM-induced PF partially via enhancing A2aR and then down-regulating TGF-β1 induced ERK1/2 signaling pathway. This will provide a significant insight of the protective effects of baicalin in PF and the underlying mechanisms may become a theoretical basis for the use of baicalin in themanagement of clinical PF.

## Abbreviations

A2aR: Adenosine A2a receptor
AECs: Alveolar epithelial cells
BLM: Bleomycin
ECM: Extracellular matrix
ELISA: Enzyme linked immunosorbent assay
EMT: Epithelial mesenchymal transition
ERK1/2: Extracellular signal regulated kinase1/2
H&E: Hematoxylin and eosin
HYP: Hydroxyproline
IOD: Integrated optical density
IPF: Idiopathic pulmonary fibrosis
KO: Knockout
MAPK: Mitogen-activated protein kinase
PAH: Pulmonary arterial hypertension
PF: Pulmonary fibrosis
TGF-β1: Transforming growth factor-β1
WT: Wild-type
